# Fang Filling

**Published:** 1860-10

**Authors:** 


*Fang Filling.*—Prof. Watt reports several cases in which the nerves of the teeth had been extirpated, and the crowns filled after his peculiar method. He claims satisfactory success. Every practitioner was compelled to allow the dead pulp to remain in a tooth before fang filling was introduced, and success frequently attended his operations. It was found, however, from many failures, injudicious practice, and the nerve was extirpated and its place partially occupied with substances used in filling; creasote being used to purify and to produce cicatrization. Greater success attending this, it was carried into a systematic operation, and when well done, is most generally highly successful. Professor Watt recommends filling the crown only with gold, after treating the pulp cavity and fang with creasote. B.

